# Supplementary material for: A Multitarget Approach to Evaluate the Efficacy of Aquilaria sinensis Flower Extract against Metabolic Syndrome
Source: Molecules. 2022 Jan 19;27(3):629. doi: 10.3390/molecules27030629 (PMC8838142; doi:10.3390/molecules27030629)

Table S1. Non-targeted analysis of methanolic extract of *A. sinensis* flower using LC-QToF.

#	RT (min)	Compound Name	Mol. formula	*m/z* [M+H]$^+$	Fragment ions	*m/z* [M-H]$^-$	Major Fragment ions
1	1.8	Malic acid	$C_4H_6O_5$	-	-	133.0140 (133.0142)	-
2	2.6	Succinic acid	$C_4H_6O_4$	-	-	117.0193 (117.0193)	73.0306
3	4.34	Phenylalanine	$C_9H_{11}NO_2$	166.0857 (166.0863)*	103.0510, 77.0368	-	-
4	5.4	Procatechuic acid (3,4-dihydroxybenzoic acid)	$C_7H_6O_4$	-	-	153.0191 (153.0193)	109.0292 [M-H-CO_2]$^-$
5	8.5	Iriflophenone 3-C-glucoside	$C_{19}H_{20}O_{10}$	409.1128 (409.1129)	247.0610 [M+H-Glc]$^+$	407.0989 (407.0984)	245.0456 [M-H-Glc]$^-$
6	8.8	Iriflophenone 3,5-di-C-β-D-glucopyranoside	$C_{25}H_{30}O_{15}$	571.1655 (571.1657)	247.0613 [M+H-2Glc]$^+$	569.1515 (569.1512)	407.0961 [M-H-Glc]$^-$ 245.0461 [M-H-2Glc]$^-$
7	11.2	Mangeferin/isomangeferin	$C_{19}H_{18}O_{11}$	423.0926 (423.0922) 445.0737 (445.0741) [M+Na]$^+$	405.0815 [M+H-H_2O]$^+$ 387.0711 [M+H-2H_2O]$^+$ 369.0589 [M+H-3H_2O]$^+$ 327.0492 [M+H-96]$^+$ 303.0503 [M+H-120]$^+$ 273.0412 [M+H-150]$^+$	421.0779 (421.0772)	403.0681 [M+H-H_2O]$^+$
8	11.75						
9	13.9	Iriflophenone 2-rhamnoside	$C_{19}H_{20}O_9$	393.1176 (393.118)	247.0611 [M+H-Rha]$^+$	391.1034 (391.1035)	245.0461 [M-H-Rha]$^-$
10	15.4	Iriflophenone 2-*O*-*a*- L-(4"-acetyl)-rhamnopyranoside /Aquilarinenoside E	$C_{21}H_{22}O_{10}$	435.1285 (435.1286)	247.0611	433.1137 (433.1140)	245.0453
11	17.2						
12	17.7						
13	20.6						
14	18.7	kaempferol-3-*O*-*β*-D-glucopyranoside	$C_{21}H_{20}O_{11}$	449.1077 (449.1078)	287.055 [M+H-Glc]$^+$	447.0931 (447.0933)	285.0395
15	23.1	Kaempferol dihexoside	$C_{27}H_{30}O_{16}$	611.1589 (611.1607)	287.055 [M+H-2Glc]$^+$	609.1451 (609.1461)	285.0402 [M-H-2Glc]$^-$
16	24.5	Aquilarisinine	$C_{25}H_{30}O_{14}$	555.1701 (555.1708)		553.1559 (553.1563)	
17	24.8	Kaempferol (*p*-coumaroyl-glucoside)/ Kaempferol (*p*-coumaroyl-galactoside)	$C_{30}H_{26}O_{13}$	595.1449 (595.1446)	287.055 [M+H-coumaroyl-Glc or Gal]$^+$	593.1307 (593.1301)	285.0399

18	25.3				147.0444 [coumaroyl+H]$^+$		
19	25.2	Isorhamnetin coumarylglucoside	$C_{31}H_{28}O_{14}$	626.1555 (625.1552)	317.0656<br>147.0441 [coumaroyl+H]$^+$	623.1403 (623.1406)	315.0505
20	26.9	Kaempferol	$C_{15}H_{10}O_6$	287.0555 (287.0550)	-	285.0410 (285.0405)	-
21	31.2	Genkwanin	$C_{16}H_{12}O_5$	285.0745 (285.0757)		283.0606 (283.0612)	
22	17.1	Aqulaside B	$C_{22}H_{22}O_{12}$	479.1181 (479.1184)	247.0609	477.1039 (477.1038)	245.0453
23	18.73	Aqulaside C (Iriflophenone derivative)	$C_{25}H_{28}O_{13}$	537.1601 (537.1603)	247.0599	535.1446 (535.1437)	245.0457
24	24.6	Aqulaside A	$C_{28}H_{26}O_{12}$	555.1491 (555.1497)	247.0606	553.1354 (553.1351)	245.0459
25	15.04	Unk-1	$C_{19}H_{26}O_{10}$	415.1591 (415.1599)		413.1460 (413.1453)	311.1139, 269.1030, 99.0452
26	12.8	Unk-2	$C_{22}H_{38}O_{12}$	-		493.2297 (493.2316)	391.1967, 349.1862, 99.0451
27	19.2	Unk-3 (Iriflophenone derivative)	$C_{18}H_{30}O_{10}$	-		405.1766 (405.1766)	245.0447

*calculated accurate mass

Figure S1: MS and MS-MS spectra of mangiferin in positive ion mode using LC-QToF

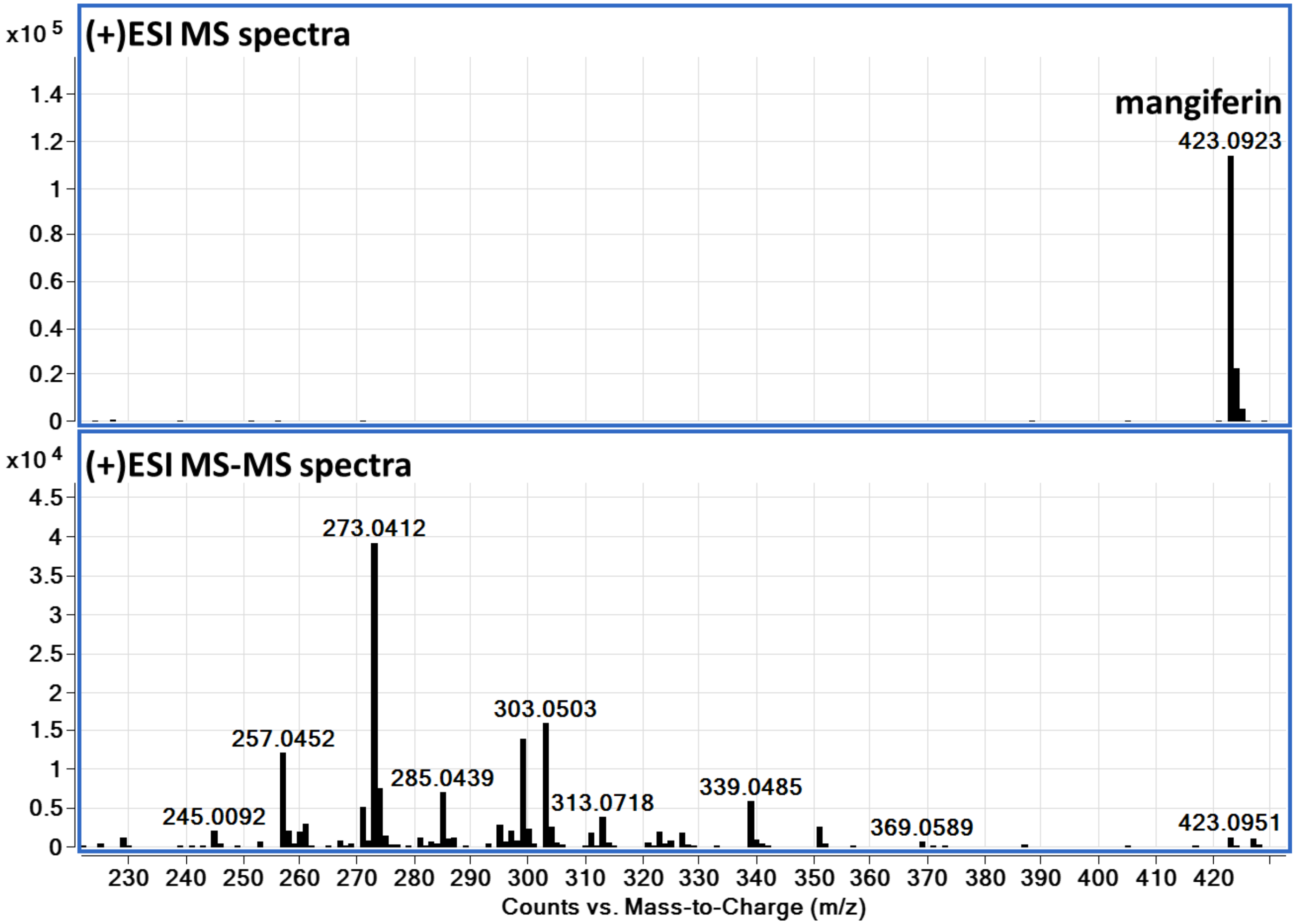

Supplement: Supplementary file 1 [file molecules-27-00629-s001.zip › molecules-1515851-supplementary.pdf]
